# Supplementary material for: Meta-analyses of Schistosoma japonicum infections in wild rodents across China over time indicates a potential challenge to the 2030 elimination targets
Source: PLoS Negl Trop Dis. 2020 Sep 2;14(9):e0008652. doi: 10.1371/journal.pntd.0008652 (PMC7491725; doi:10.1371/journal.pntd.0008652)
Supplement: S1 Table — (DOCX) [file pntd.0008652.s002.docx]

**S1 Table. *Schistosoma japonicum* infections in different species of rodents from 13 articles**

| **Author and year of publication*** | **Species of rodents** |  | **No. of rodents examined** | **No. of rodents infected** | **Prevalence（%）** |
| --- | --- | --- | --- | --- | --- |
| Lu, 2019[[1](#_ENREF_1)] | *Rattus norvegicus* |  | 67 | 4 | 5.97 |
|  | *Rattus flavipectus* |  | 54 | 1 | 1.85 |
|  | *Apodemus agrarius* |  | 14 | 0 | 0 |
|  | *Microtus fortis* |  | 20 | 0 | 0 |
|  | *Microtus montanus* |  | 14 | 0 | 0 |
|  | *Suncus murinus* |  | 3 | 0 | 0 |
| Van Dorssen 2017[[2](#_ENREF_2)] | *Rattus norvegicus*  *cus* |  | 37 | 0 | 0 |
|  | *Microtus fortis* |  | 46 | 0 | 0 |
| Wang 2018[[3](#_ENREF_3)] | *Apodemus agrarius* |  | 66 | 0 | 0 |
| Lu 2018[[4](#_ENREF_4)] | *Rattus norvegicus* |  | 16 | 0 | 0 |
| Shao 2016[[5](#_ENREF_5)] | *Rattus sladeni* |  | 165 | 1 | 0.61 |
|  | *Rattus norvegicus* |  | 25 | 0 | 0 |
|  | *Rattus tanezumi* |  | 9 | 0 | 0 |
|  | *Apodemus chevrier* |  | 62 | 0 | 0 |
| Luo 2014[[6](#_ENREF_6)] | *Apodemus agrarius* |  | 34 | 0 | 0 |
| Guo1 2013[[7](#_ENREF_7)] | *Rattus flavipectus* |  | 7 | 1 | 14.3 |
|  | *Rattus norvegicus* |  | 11 | 1 | 9.1 |
|  | *Rattus rattus* |  | 22 | 5 | 22.7 |
|  | *Microtus fortis* |  | 11 | 0 | 0 |
| Guo2 2013[[7](#_ENREF_7)] | *Rattus flavipectus* |  | 10 | 1 | 10 |
|  | *Rattus norvegicus* |  | 16 | 1 | 6.3 |
|  | *Rattus rattus* |  | 10 | 0 | 0 |
|  | *Microtus fortis* |  | 15 | 0 | 0 |
| Guo3 2013[[7](#_ENREF_7)] | *Rattus flavipectus* |  | 11 | 2 | 18.2 |
|  | *Rattus norvegicus* |  | 5 | 1 | 20 |
|  | *Rattus rattus* |  | 7 | 2 | 28.6 |
|  | *Microtus fortis* |  | 16 | 0 | 0 |
| Guo4 2013[[7](#_ENREF_7)] | *Rattus flavipectus* |  | 4 | 0 | 0 |
|  | *Rattus norvegicus* |  | 9 | 2 | 22.2 |
|  | *Rattus rattus* |  | 2 | 0 | 0 |
|  | *Microtus fortis* |  | 4 | 0 | 0 |
| Shao 2011[[8](#_ENREF_8)] | *Rattus sladeni* |  | 80 | 0 | 0 |
|  | *Apodemus chevrier* |  | 52 | 0 | 0 |
|  | *Rattus norvegicus* |  | 20 | 0 | 0 |
|  | *Rattus tanezumi* |  | 5 | 0 | 0 |
| Ding 2008[[9](#_ENREF_9)] | *Rattus tanezumi* |  | 65 | 7 | 10.77 |
|  | *Rattus norvegicus* |  | 73 | 14 | 19.18 |
|  | *Rattus losea* |  | 41 | 4 | 9.76 |
|  | *Rattus rattus* |  | 37 | 5 | 13.51 |
| YangW 1999[[10](#_ENREF_10)] | *Rattus norvegicus* |  | 343 | 1 | 0.29 |
|  | *Rattus flavipectus* |  | 1163 | 1 | 0.09 |
|  | *Rattus nitidus* |  | 237 | 0 | 0 |
|  | *Rattus sladeni* |  | 57 | 1 | 1.75 |
|  | *Apodemus chevrier* |  | 29 | 0 | 0 |
|  | *Eothenomys miletus* |  | 16 | 0 | 0 |
|  | *Rattus koraten* |  | 6 | 0 | 0 |
|  | *Mus pahari* |  | 2 | 0 | 0 |
|  | *Mus musculus* |  | 4 | 0 | 0 |
|  | *Mus caroli* |  | 1 | 0 | 0 |
|  | *Mus pahari* |  | 2 | 0 | 0 |
|  | *Micromys minutus* |  | 6 | 0 | 0 |
| Xu 1999[[11](#_ENREF_11)] | *Rattus norvegicus* |  | 189 | 113 | 59.8 |
| Zhou1 1996[[12](#_ENREF_12)] | *Rattus norvegicus* |  | 230 | 13 | 5.65 |
|  | *Rattus flavipectus* |  | 52 | 0 | 0 |
|  | *Apodemus agrarius* |  | 37 | 3 | 8.11 |
|  | *Microtus fortis* |  | 86 | 0 | 0 |
| Zhou2 1996[[12](#_ENREF_12)] | *Rattus norvegicus* |  | 185 | 0 | 0 |
|  | *Apodemus agrarius* |  | 45 | 0 | 0 |
| Xu 1995[[13](#_ENREF_13)] | *Apodemus sylvaticus* |  | 115 | 1 | 0.87 |

Reference

1. Lu SB, Chen NG, Liu YM, Zhou LY, Wang YS, et al. (2019) Survey of *Schistosoma japonicum* infections in wild animals in hilly transmission-controlled areas of Jiangxi province. Chin J Schisto Control 31: 463-467.

2. Van Dorssen CF, Gordon CA, Li Y, Williams GM, Wang Y, et al. (2017) Rodents, goats and dogs - their potential roles in the transmission of schistosomiasis in China. Parasitology 144: 1633-1642.

3. Wang H, Xiong YL, Zhang JJ, Li Y, Zuo YT, et al. (2018) Assessment of schistosomiasis transmission risk after flood damage in Wuhan city. Chin J Schisto Control 30: 410-414.

4. Lu C, Zhou LY, Xing XY, Lin DD, Chen T, et al. (2018) Analysis of transmission risk factors of schistosomiasis in a hilly demonstration plot of transmission interruption. Chin J Parasitol Parasit Dis 36: 333-339.

5. Shao ZT, Feng XG, Dong Y, Xiong MT, Shi XW (2016) Investigation on the infection of schistosomiasis in small mammals after achieving control standards in Eryuan county, Yunnan province. Chin J Vector Biol & Control 27: 474-477.

6. Luo HT, Wang H, Xu MX, Zhou SM (2014) Investigation on the infection of *Schistosoma japonicum* in Wuhan section of the Yangtze River. J Med Pest Control 30: 106-107.

7. Guo Y, Jiang M, Gu L, Qiao Y, Li W (2013) Prevalence of *Schistosoma japonicum* in wild rodents in five islands of the West Dongting lake, China. J Parasitol 99: 706-707.

8. Shao ZT, Feng XG, Dong Y, Xiong MT (2011) Investigation on the infectious source of schistosomiasis japonica in Eryuan county, Yunnan province. J Trop Dis Parasitol 9: 138-142, 160.

9. Ding XJ, Zhao HM, Zhao SY (2008) Investigation on the natural infection of wild rodents with *Schistosoma japonicum* in Jingzhou city, Hubei province. Progress in Veterinary Medicine: 14-17.

10. Yang GR, Wu X, Xiong MT, Fan CZ, Tao KH (1999) Role of rodents in transmitting schistosomiasis in the Plateau Plains. Chin J Vector Bio & Control: 449-453.

11. Xu GY, Tian JC, Chen GM, Yang HM, Qiu L (1999) Study on the plague source of *Schistosoma japonicum* ditch in Nanjing city. Journal of Practical Parasitic Diseases: 6-8.

12. Zhou PS, Ke CL, Lin LH, Huang JL (1996) Study on integrated control measures and effects of schistosomiasis in a farm in Hunan province. Literatue and Information on Preventine Medicine: 1-2.

13. Xu FS, Gu XG, Zhao WX, Li YX, Yin HZ (1995) Role of different sources of infection in transmission of schistosomiasis in mountainous areas. Journal of Practical Parasitic Diseases: 129.
