# Supplementary figures and images for: Meta-analyses of Schistosoma japonicum infections in wild rodents across China over time indicates a potential challenge to the 2030 elimination targets
Source: PLoS Negl Trop Dis. 2020 Sep 2;14(9):e0008652. doi: 10.1371/journal.pntd.0008652 (PMC7491725; doi:10.1371/journal.pntd.0008652)

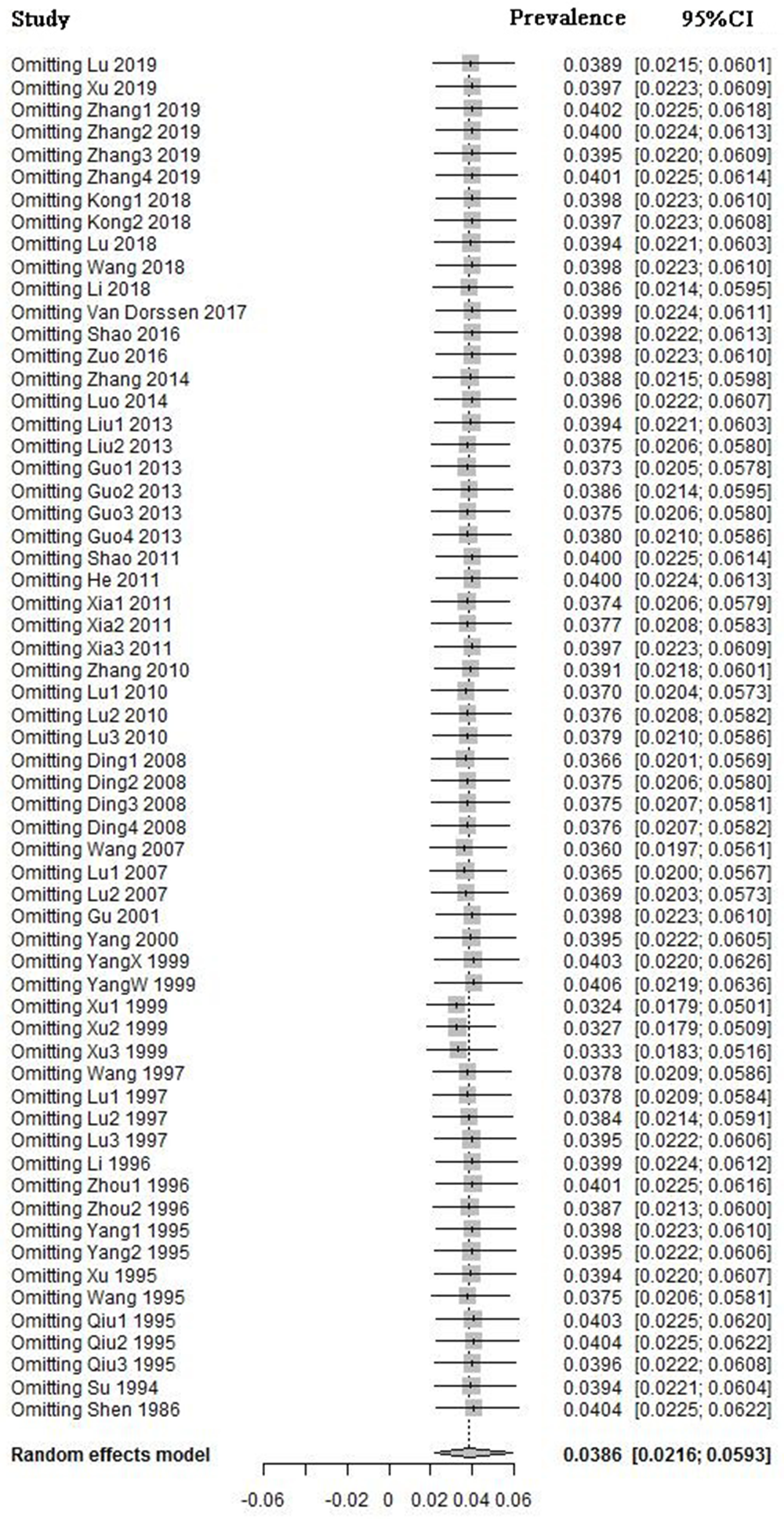

Supplement: S1 Fig — (TIF) [file pntd.0008652.s003.tif]
