# Supplementary material for: Meta-analyses of Schistosoma japonicum infections in wild rodents across China over time indicates a potential challenge to the 2030 elimination targets
Source: PLoS Negl Trop Dis. 2020 Sep 2;14(9):e0008652. doi: 10.1371/journal.pntd.0008652 (PMC7491725; doi:10.1371/journal.pntd.0008652)
Supplement: Attachment — Submitted filename: V3_Responses to Reviewers comments_Clean.docx [file pntd.0008652.s004.docx]

Dear Prof. Justin V. Remais,

Thank you very much indeed for the comments and suggestions on the manuscript (No. PNTD-D-19-01947) from both yourselves and the three referees, all of which we found very helpful and have been fully incorporated into our further revised text - precise of which are also provided below. We hope our manuscript is now suitable for publication in *PLoS Neglected Tropical Diseases* and we very much look forward to hearing from you.

Kindest regards,

Prof. Da-Bing Lu, on behalf of all the authors

Reviewer's Responses to Questions

<b>Key Review Criteria Required for Acceptance?</b></br></br>

As you describe the new analyses required for acceptance, please consider the following:</br></br>

<b>Methods</b></br></br>

-Are the objectives of the study clearly articulated with a clear testable hypothesis stated?</br>

-Is the study design appropriate to address the stated objectives?</br>

-Is the population clearly described and appropriate for the hypothesis being tested?</br>

-Is the sample size sufficient to ensure adequate power to address the hypothesis being tested?</br>

-Were correct statistical analysis used to support conclusions?</br>

-Are there concerns about ethical or regulatory requirements being met?</br></br>

Reviewer #1: (No Response)

Reviewer #2: This study systematically analyzed *S. japonicum* infections in wild rodents from the published literature over the last forty years after the introduction of praziquantel for schistosomiasis treatment in humans and bovines in 1980s. The analysis seems appropriate.

Answers: Thank you very much for your positive comments.

Reviewer #3: (No Response)

--------------------

<b>Results</b></br></br>

-Does the analysis presented match the analysis plan?</br>

-Are the results clearly and completely presented?</br>

-Are the figures (Tables, Images) of sufficient quality for clarity?</br></br>

Reviewer #1: (No Response)

Reviewer #2: Although numbers of schistosomiasis cases in humans and bovines have been greatly reduced in China, change of infection prevalence in rodents differed among provinces or eco-epidemiology settings.

In Table 1, the author and year of publication do not match for many of the studies highlighted.

Answers: Agreed and amended - we have corrected errors in abbreviations for Chinese names in cited articles (please see References and Table 1).

Reviewer #3: (No Response)

--------------------

<b>Conclusions</b></br></br>

-Are the conclusions supported by the data presented?</br>

-Are the limitations of analysis clearly described?</br>

-Do the authors discuss how these data can be helpful to advance our understanding of the topic under study?</br>

-Is public health relevance addressed?</br></br>

Reviewer #1: (No Response)

Reviewer #2: While China is determining to achieve the target of complete interruption of zoonotic schistosomiasis in the country as a whole by 2030, the authors conclude that enrolling an investigation of S. japonicum infections in small animal rodents into the ongoing monitoring and evaluation program is essential to determine whether local transmission is ongoing.

There are a number of issues that the authors need to consider carefully as their conclusions as presented are somewhat misleading and could impact negatively (although unlikely as the programme is well advanced) on the national control programme for schistosomiasis in China.

Answers: Thank you very much for your comments and very helpful suggestions. It is well known that great achievement in schistosomiasis control has been made in China. However, to fulfill the 2030 target for elimination/complete interruption of all potential ongoing schistosomiasis transmission to humans, and in order to achieve WHO validation/verification, it is imperative that adequate surveillance and monitoring across all potential definitive hosts must be performed. Indeed this may be particularly pertinent for *S. japonicum*, where at least 46 mammalian species have already been demonstrated to serve as reservoirs for *S. japonicum*, with bovines and in particular rodents now proving key hosts in many regions [1,2]. Thus we strongly propose here that the human-centric (and admittedly to an increasing extent molluscan-centric) criteria is no longer sufficient as the global goals move from control to elimination [3]. In contrast, as we illustrate here, infections in wildlife, in particular that amongst the most abundant of these being small rodents, long-since neglected, may prove to be critical factors in the ongoing transmission of schistosomiasis in both China [1,2], our focus here, but also beyond [4-6].

We do find these referee’s suggestions thus highly useful and highly pertinent and have further clarified these points within our revised text. For example, we have modified our conclusion as ‘enrolling an investigation of *S. japonicum* infections in small animal rodents **in the endemic areas, where infections are no longer found in humans or domestic animals**, into the ongoing monitoring and evaluation programs aimed to document ‘interruption of transmission’ are essential’.

References:

1. Liu XP, Wang TP, Wang QZ, Yin XM (2013) Infection status of sources of schistosomiasis japonica in marshland and hilly regions. Journal of Pathogen Biology 8: 445-447, 410.

2. Rudge JW, Webster JP, Lu DB, Wang TP, Fang GR, et al. (2013) Identifying host species driving transmission of schistosomiasis japonica, a multihost parasite system, in China. Proc Natl Acad Sci U S A 110: 11457-11462.

3. Xu J, Li SZ, Chen JX, Wen LY, Zhou XN (2017) Playing the guiding roles of national criteria and precisely eliminating schistosomiasis in P. R. China. Chin J Schisto Control 29: 1-4.

4. Catalano S, Sene M, Diouf ND, Fall CB, Borlase A, et al. (2018) Rodents as Natural Hosts of Zoonotic *Schistosoma* Species and Hybrids: An Epidemiological and Evolutionary Perspective From West Africa. J Infect Dis 218: 429-433.

5. Deol AK, French MD, Webster JP (2020) Schistosomiasis and the Global Goals. New England Journal of Medicine: In press.

6. Catalano S, Leger E, Fall C-B, Borlase A, Diop SD, et al. (2020) Multi-host transmission of Schistosoma mansoni in Senegal. Emerging Infectious Diseases: In press.

It is not clear how prevalence was obtained - dissection of animals or detection of eggs in faeces.

Answers: We have now added the information on detection methods. See Table 1.

There are a number of rodent species in China and it would be important to include these if known from the studies considered as different species of rodents range from fully permissive to non-permissive for schistosome infections. Similarly it is unclear if any of the quoted studies include intensity information which is unfortunate as the Index of Potential Contamination (Vercruysse et al. Trends Parasitol. 17, 256-61, 2001) (important for defining schistosome transmission parameters) cannot be calculated. A recent article by Van Dorssen et al. Parasitology 2017, 144: 1633-42) showed that the Contamination index for rodents (two species) in the Dongting Lake area of Hunan province was extremely low.

Answers: Many thanks for your critical comments. We have carefully read all included publications and found only 13 retrieved papers with 17 studies provided this aspect of information on rodent species investigated. We have here listed the reported infection prevalence among rodent species (please see Table 4). A total of 19 rodent species were investigated and *S. japonicum* infections were found in seven rodent species. The *Rattus norvegicus* and *Rattus flavipectus* were the most common species investigated. The top five infection prevalence ranged from 10.68% in *Rattus rattus* to 0.87% in *Apodemus sylvaticus*. No infections were found in three species (i.e. *Apodemus chevrier*, *Microtus fortis* and *Rattus nitidus*) whose pooled sample size each were over 100. Among them *Microtus fortis* is the only known mammalian host in which schistosomes of *S. japonicum* are unable to mature and cause significant pathogenesis. We have incorporated these details into our revised text. Please see Table 4, and sections of Method, Results and Discussion.

Indeed we agree that the Contamination Index can be an important measure in **comparing the role of different reservoirs species or final host individuals in transmission**. The paper by Van Dorssen et al (2017) is extremely interesting and we have included it in our meta-analysis of pooled prevalence (see Ref 42). We accept that, in comparison with humans or large domestic animals, for instance, an individual rodent excretes a much smaller portion of feces a day, purportedly leading to a smaller value of the index. Furthermore, unfortunately, there are currently few papers providing direct information on infection intensities amongst non-human definitive hosts and/or specifically including the Contamination Index. We thereby raise the potential caveat within our revised text (e.g. see limitations within Discussion). Nevertheless, of those publications that do include *S. japonicum* infection intensities, significantly higher mean infection intensities have been documented for rodents as compared to humans and domestic livestock within China [7]. Furthermore, the commensal and sympatric nature of rodents (literally meaning they ‘share our table’ places them in close human contact, with the widespread distribution of rodent feces around ditches and snail habitats place them in ideal locations for viable hatching and subsequent ongoing transmission. This relative importance of rodents has already been demonstrated for other multi-host systems of major human and animal importance, such as through both epidemiological surveys and predictive mathematical modelling for bTB transmission [8]. Furthermore, for schistosomiasis, previous work has shown it is the same genotypes circulating through rodents and humans for both *S. japonicum* in China [9] and the Philippines [10] as well as for other species, such as *S. mansoni* in Africa [6].

As many previously endemic areas across China (and beyond) are now reporting little or no infections in humans or domestic animals, before elimination can be declared, it is imperative to determine alternative key reservoir hosts that may serve to continue transmission, even if by only occasional spillover, and thereby facilitate the potential subsequent re-emergence/bounce back (via infected snails).

References:

7. Lu DB, Wang TP, Rudge JW, Donnelly CA, Fang GR, et al. (2010) Contrasting reservoirs for *Schistosoma japonicum* between marshland and hilly regions in Anhui, China-a two-year longitudinal parasitological survey. Parasitology 137: 99-110.

8. Mathews F, Macdonald DW, Taylor GM, Gelling M, Norman RA, et al. (2006) Bovine tuberculosis (Mycobacterium bovis) in British farmland wildlife: the importance to agriculture. Proc Biol Sci 273: 357-365.

9. Lu DB, Wang TP, Rudge JW, Donnelly CA, Fang GR, et al. (2009) Evolution in a multi-host parasite: Chronobiological circadian rhythm and population genetics of Schistosoma japonicum cercariae indicates contrasting definitive host reservoirs by habitat. Int J Parasitol 39: 1581-1588.

10. Rudge JW, Carabin H, Balolong E, Tallo V, Shrivastava J, et al. (2008) Population genetics of *Schistosoma japonicum* within the Philippines suggest high levels of transmission between humans and dogs. PLoS Neglected Tropical Diseases 2: e340.

Furthermore, the authors do not provide any information for any of the studies on the maturity of worms in infected rodents or the viability of eggs other than quoting an older article by Mao (1990) stating "The egg hatching rate from an infected rodent's feces was 14.5 times that of human feces". This statement needs to be revisited as it does not seem to be realistic and needs additional experimental evidence in support, particularly as several different rodent species, with different susceptibilities to infection, will be present in the endemic areas.

Answers: Thank you for this and we have both rephrased, and expanded upon this potential issue within our revised text. We acknowledge that many of the past publications investigating *S. japonicum* in wild rodents in China have usually relied upon lethal traps or live traps before humane euthanasia, and infection status determined following perfusion and/or dissection to identify schistosome adult worms and/or egg granuloma on livers, the latter of which is obvious under a microscope, rather than hatching eggs from rodent stool directly (Table 1).

It may be nonetheless worthy to note again the indirect measures of extremely high prevalence and/or indeed infection intensities in rodents relative to other potential definitive hosts, particularly in highland regions of China [7,11], and likewise the Philippines [10,12]. Similarly, cercarial shedding from intermediate host snails has been reported to be significantly higher in those Chinese regions where rodents appear to be key hosts for maintaining transmission relative to other potential human and animal host species [11,13], as well at their shedding chronobiology [9]. Furthermore, recent work on other intestinal schistosome species transmitted by rodents also show strong associations between infection prevalence and intensities as identified by adult worm to egg ratios and across contrasting diagnostic methodologies (e.g.[14]). We have expanded our revised text to clarify this. Similarly we stress that further work is now imperative to fully evaluate the hatching rates/viability of schistosomes shed from rodents across both China and beyond.

References:

11. Rudge JW, Lu DB, Fang GR, Wang TP, Basanez MG, et al. (2009) Parasite genetic differentiation by habitat type and host species: molecular epidemiology of *Schistosoma japonicum* in hilly and marshland areas of Anhui Province, China. Mol Ecol 18: 2134-2147.

12. Fernandez TJ, Jr., Tarafder MR, Balolong E, Jr., Joseph L, Willingham Iii AL, et al. (2007) Prevalence of schistosoma japonicum infection among animals in fifty villages of samar province, the Philippines. Vector Borne Zoonotic Dis 7: 147-156.

13. Lu DB, Rudge JW, Wang TP, Donnelly CA, Fang GR, et al. (2010) Transmission of *Schistosoma japonicum* in marshland and hilly regions of China: parasite population genetic and sibship structure. PLoS Negl Trop Dis 4: e781.

14. Catalano S, Symeou A, Marsh KJ, Borlase A, Leger E, et al. (2019) Mini-FLOTAC as an alternative, non-invasive diagnostic tool for *Schistosoma mansoni* and other trematode infections in wildlife reservoirs. Parasit Vectors 12: 439.

Reviewer #3: (No Response)

--------------------

<b>Editorial and Data Presentation Modifications?</b></br><br/>

Use this section for editorial suggestions as well as relatively minor modifications of existing data that would enhance clarity. If the only modifications needed are minor and/or editorial, you may wish to recommend “Minor Revision” or “Accept”.

Reviewer #1: (No Response)

Reviewer #2: English style and grammar needs considerable attention. Praziquantel is misspelt on numerous occasions. In Table 1, the author and year of publication do not match for many of the studies highlighted. References do not conform to the correct journal style.

Answers: Agreed and amended throughout.

Reviewer #3: (No Response)

--------------------

<b>Summary and General Comments</b></br></br>

Use this section to provide overall comments, discuss strengths/weaknesses of the study, novelty, significance, general execution and scholarship. You may also include additional comments for the author, including concerns about dual publication, research ethics, or publication ethics. If requesting major revision, please articulate the new experiments that are needed.

Reviewer #1: Needs to be checked for English. Perhaps the fourth author, Joanne, could do this.

Answers: Our text has now been fully revised.

Line 65: seemed is past tense, presumably it is imperative

Answers: We have agreed and amended.

Line 70: transmission interruption (and would a lay person know what this means?)

Answers: We have now included definitions for such key terms (See the first paragraph of Introduction).

Line 209: Differences in how infection status was evaluated?

Answers: The infection status was evaluated with dissection and fecal examination, and we have now clarified and expanded upon these details within our revised text. See Table 1.

Line 230: Space before (

Answers: Agreed and amended.

There have been several papers in the past throwing doubt as to the permissibility of rodents as schistosome hosts – i.e. that they do not have viable infections. Any comment on this? Were viability measures included in the studies examined? What methods were used to determine infection? Eggs in stool? Conceivably rodents could be consuming the eggs in the faeces of humans or other animals and passing them through the stool?

Answers: As for Referee 1’s query above, we acknowledge that, although standardized data remain lacking, some species of rodents, for example *Microtus fortis*, have been reported to be not permissible to *S. japonicum* [15]. It has been proposed that the growth retardation and pathological changes observed in schistosomula from *M. fortis* might be the result of apoptosis and the differential expression of some apoptotic, developmental, metabolic and signal transduction-related genes. These, in turn, would adversely affect the survival and development of *S. japonicum* in *M. fortis*, and lead ultimately to the death of the migrating schistosomula [16]. However, the exact mechanism underlying remains unclear.

Wang et al [17] showed in the laboratory that *S. japonicum* eggs could survive, and indeed remain viable, following passage through dogs and chickens and suggested both dogs and chickens can serve as potential transport hosts for (rather than and/or in addition to reservoir hosts for) *S. japonicum* in endemic areas. We have now added additional information on detection methods used within the literature cited. As seen in Table 1, the dissection approach for worms or liver granulomas were used in almost all studies.

References:

15. Wu K (1957) Schistosomiasis japonica among domestic and wild animals in China. Chin Vet J 3: 98-100.

16. Peng J, Gobert GN, Hong Y, Jiang W, Han H, et al. (2011) Apoptosis governs the elimination of *Schistosoma japonicum* from the non-permissive host *Microtus fortis*. PLoS One 6: e21109.

17. Wang T, Wang F, Zhu L, Zhang S, Ornbjerg N, et al. (2006) *Schistosoma japonicum* eggs survive passage through dogs and chickens. Vet Parasitol 140: 362-365.

I am aware of at least one paper on rodents in China that has not been included; Van Dorssen et al 2017, Para. 144 (12):163-1642.

Answers: Many thanks, and as for Referee 1 above, the excellent work by Van Dorssen et al (2017) has now been included in our meta-analysis. See Ref 42. Likewise, further key papers relating to rodent transmission of schistosomiasis in China and beyond have been incorporated into our revised text.

Do you think your search terms were too restrictive and a more open ended search would have yielded additional papers?

Answers: On the basis of this valid suggestion, we subsequently modified the term and updated our search throughout to December of 2019. Four publications, including Van Dorssen et al 2017, have been included in our meta-analysis (See within the sub-section of Search Strategy and Figure 1 for details), together with broader recent references on schistosomiasis in wild rodents in general have now been incorporated into our revised text.

Reviewer #2: This authors need to temper their conclusions in light of the review comments made above.

Answers: Many thanks. We agreed and have amended. See sub-sections of Summary and Conclusion.

The title of the article does not convey the true content of the paper.

Answers: We have revised it. See Title.

Reviewer #3: This study tried to estimate the infection prevalence of S. japonicum in rodents in China by meta-analysis, and it is interesting. However, people used to pay attention to infections in humans and livestock, and in snail intermediate hosts, and to ignore S. japonicum infections in rodents or other wild animals. As a result, there might be not enough studies to reflect the epidemic situation of rodents in China. As the author said, few publications on studies were performed in Jiangxi and Sichuan, two of the most serious endemic provinces.

Hence, caution is needed in making conclusions. For example, in Table 3, the prevalence of rodents in hilly and mountainous regions was 0 during 1980-2003. Is it really the case? And is it real that ‘the estimate significantly increased in the hilly and mountainous regions’. There is also no data to support that “The estimate significantly decreased over time in the marshland and lakes”, for the 95% CI of prevalence during 1980-2003 is (0.0164, 0.1999), and that during 2004-2018 is (0.0145, 0.0834) (Table 3).

Answers: Thank you very much for both your positive and critical comments. We admit that in hilly and mountainous regions only 8 studies performed during 1980 to 2003 were included. The number was lower compared to a total of 20 studies during 2004 to 2018. We have added a possible explanation for the change in the estimate (see Discussion). We have also updated our search and re-analyzed the data accordingly. The estimate decreased but not significantly over time in the marshland and lakes (see Table 3). The results from this work thus showed a high infection prevalence of 3.86% in wild rodents, with no apparent change before and after 2004.

However, even given the caveats acknowledged in terms for the literature available to date, this does not detract at all from the fundamental message here that the role of wild rodents in the transmission of schistosomiasis must not be ignored. Furthermore, given the upcoming revised WHO guidelines and the move of global targets from schistosomiasis disease control to full elimination/interruption of transmission, now it is the critical time to begin to examine the potential role of rodent wildlife reservoirs for ongoing transmission within China and across Asia, as well as globally.
