# Supplementary material for: Meta-analyses of Schistosoma japonicum infections in wild rodents across China over time indicates a potential challenge to the 2030 elimination targets
Source: PLoS Negl Trop Dis. 2020 Sep 2;14(9):e0008652. doi: 10.1371/journal.pntd.0008652 (PMC7491725; doi:10.1371/journal.pntd.0008652)
Supplement: Attachment — Submitted filename: V4 Reponses to Reviewers comments_Submitted.docx [file pntd.0008652.s005.docx]

Dear Prof. Darren J. Gray and Prof. Justin Remais,

Thank you and your two referees very much indeed for the comments and suggestions on our manuscript (No. **PNTD-D-19-01947R1** entitled ‘Meta-analyses of *Schistosoma japonicum* infections in wild rodents across China over time indicates a potential challenge to the 2030 elimination targets’), all of which we found very helpful and have been incorporated into our further revised text – precise details of which are provided in our *Responses to Referees* below. As suggested, we have now tuned down our main conclusions, including the manuscript title itself. Likewise we have further clarified the limitations/limited number of currently available published studies and hence how we hope to help stimulate future research and monitoring in this area to both enable robust conclusions to be drawn (and if thereby deemed necessary, formalized monitoring amongst rodent wildlife populations to be implemented).

However, given the keen current interest of the potential role of rodent reservoirs for ongoing/re-emerging schistosomiasis transmission in general, and by the WHO in terms of their revised 2030 Guidelines, as well as pending validation/verification of interruption of transmission in particular, we do still feel it imperative to raise their potential importance here. We have therefore provided further evidence here in support of their role (citing, for example, viable eggs/miracida and matching genotypic/phylogenetic data from sympatric humans and rodents). We are of course fully mindful that consideration of a potential wildlife reservoir of infection will not be easily accepted, given the challenges this raises to verification of elimination. However, we also feel that this must be balanced with the available evidence base to date and, as mentioned, help stimulate the need for future research.

We thus hope our re-revised, and appropriately modified/re-toned, manuscript is now suitable for publication in *PLoS Neglected Tropical Diseases* and we very much look forward to hearing from you.

Kindest regards,

Prof. Da-Bing Lu, on behalf of all the authors

Reviewer's Responses to Questions

<b>Key Review Criteria Required for Acceptance?</b></br></br>

As you describe the new analyses required for acceptance, please consider the following:</br></br>

<b>Methods</b></br></br>

-Are the objectives of the study clearly articulated with a clear testable hypothesis stated?</br>

-Is the study design appropriate to address the stated objectives?</br>

-Is the population clearly described and appropriate for the hypothesis being tested?</br>

-Is the sample size sufficient to ensure adequate power to address the hypothesis being tested?</br>

-Were correct statistical analysis used to support conclusions?</br>

-Are there concerns about ethical or regulatory requirements being met?</br></br>

Reviewer #1: (No Response)

Reviewer #2: (No Response)

--------------------

<b>Results</b></br></br>

-Does the analysis presented match the analysis plan?</br>

-Are the results clearly and completely presented?</br>

-Are the figures (Tables, Images) of sufficient quality for clarity?</br></br>

Reviewer #1: (No Response)

Reviewer #2: (No Response)

--------------------

<b>Conclusions</b></br></br>

-Are the conclusions supported by the data presented?</br>

-Are the limitations of analysis clearly described?</br>

-Do the authors discuss how these data can be helpful to advance our understanding of the topic under study?</br>

-Is public health relevance addressed?</br></br>

Reviewer #1: (No Response)

Reviewer #2: (No Response)

--------------------

<b>Editorial and Data Presentation Modifications?</b></br><br/>

Use this section for editorial suggestions as well as relatively minor modifications of existing data that would enhance clarity. If the only modifications needed are minor and/or editorial, you may wish to recommend “Minor Revision” or “Accept”.

Reviewer #1: (No Response)

Reviewer #2: (No Response)

--------------------

<b>Summary and General Comments</b></br></br>

Use this section to provide overall comments, discuss strengths/weaknesses of the study, novelty, significance, general execution and scholarship. You may also include additional comments for the author, including concerns about dual publication, research ethics, or publication ethics. If requesting major revision, please articulate the new experiments that are needed.

Reviewer #1: English is much improved, some small issues remain. See specific comments.

Answer: Thank you very much for your positive comments and careful reading.

Abstract

Line 23: This first sentence can be worded much better.

Answer: Agreed and amended. Please see Line 24.

Line 45: ‘change was observed between before and after 2004’ – what does this mean? Between 2004 and what? No significant changes were observed, ever?

Answer: We have rephrased our text to clarify accordingly. Please see Line 49-50.

Line 45: estimated

Answer: Agreed and amended. Please see Line 50.

Line 58: Are there population estimates available for rodents, or notes on plague years? Obviously very few papers on rodents and schisto, so linking to total populations in endemic areas might be useful.

Answer: This is a very interesting point, thank you. We have looked for appropriate publications on rodent populations in China, but unfortunately none appeared to be available. We have therefore included some text in our revised discussion regarding the importance of future work estimating and monitoring rodent populations within such disease-endemic regions.

Line 79: delete often and levels

Answer: Agreed and amended. Please see Line 85.

Line 80: has instead of have, and in instead of amongst

Answer: Agreed and amended. Please see Line 86.

Line 81: Delete ‘of the few performed to date’

Answer: Agreed and amended. Please see Line 87.

Line 84: that instead of the

Answer: Agreed and amended. Please see Line 91.

Line 94: Ah, so only papers where dissection occurred? Any papers identified but not used that did not use dissection, but other methods?

Answer: We apologize for the confusion here. With the exception of two papers not reporting the detail method used, almost all papers reported that dissection were used. Please see Table 1. We have clarified our text accordingly. Please see Line 101.

Line 96: ‘change was observed between before and after 2004’ – what does this mean? Between 2004 and what? No significant changes were observed, ever?

Answer: We have clarified our text accordingly. Please see Line 103-104.

Line 112: After Schistosoma in full for mansoni, can abbreviate to S. haematobium, and S. japonicum

Answer: Agreed and amended. Please see Line 129.

Line 114: This sentence is awkward, needs to be re-written

Answer: Agreed and amended. Please see Line 131.

Line 115: A the beginning of the national

Answer: Agreed and amended. Please see Line 132.

Line 122: Delete for example

Answer: Agreed and deleted. Please see Line 139.

Line 150: provide

Answer: Thank you very much. We have amended. Please see Line 170.

Line 158: Whilst

Answer: We have amended. Please see Line 179.

Line 177: papers

Answer: We have amended. Please see Line 200.

Line 194: investigations

Answer: We have amended. Please see Line 217.

Line 258: add the p value in brackets after significantly

Answer: Agreed and added. Please see Lines 282, 284, 286 and 290.

Line 269: ordered by year of publication

Answer: Agreed and amended. Please see Line 295.

Line 312-318 is a loooong sentence. I would put the second part of the sentence first, then follow up with, this is consistent blah blah blah, first part of sentence.

“Estimates from lake and marshland regions showed a non-significant decline following the increased control pressures imposed post 2004, whereas infection prevalence levels in rodents

significantly increased within the hilly and mountainous regions. This is consistent with mathematical models identifying rodents as current key hosts responsible for maintaining S. japonicum transmission within hilly/mountainous regions, with bovines as key hosts only within lake and marshland regions.”

Answer: Agreed and amended. Please see Line 339-346.

Line 338: Definitely need to make the point of discrepancy’s in studies performed. Looking at table 3, Jiangsu 1980-2003 6 papers, 2004-2018 2. Not a lot of available data to make too many sweeping statements on prevalence increases and decreases without referencing that there is little info available. You then have a different bias in Hubei with 11 studies post-204, and only 2 pre 2004. Obviously it is what it is, but it needs to be acknowledged here.

Answer: We have agreed and have now acknowledged accordingly. Please see Line 385-387.

Line 318: identified very few

Answer: Agreed and amended. Please see Line 419.

Table 3: May be useful to put the number of rodents in. Particularly when looking at the prev. I noticed looking at tbale 2 that while there were more studies in 2004-2018 than pre-2004, there were less rodents examined. So that is important info to have in table 3 for this comparison as well.

Answer: Thank you very much. We have added the information on numbers of pooled rodents for each subgroup. Please see Table 3. In addition, we have added the values of statistics and P when comparing subgroups. See Table 2 & 3.

Reviewer #2: I appreciate the amount of work and effort that the authors have undertaken but this reviewer is still not convinced about the major conclusion that wild rodents contribute substantially to transmission of S. japonicum and that they will be a major barrier preventing 2030 elimination targets in China. The message presented is too strong and this reviewer does not subscribe to their conclusions for the earlier reasons I presented. Table 1 provides useful information but again there is no detail on the viability of eggs in the infected animals. It is also unclear what the % density of rodents means.

Answer: Thank you very much for both your positive and critical comments here. Dissection was most frequently used for identification of schistosome infections because egg granuloma of livers, under a microscope, is much clearer and the viable eggs are distinct for an examiner working on schistosomiasis. In our field work performed during 2006-2007 in China, we sampled miracidia (hatched from viable eggs in water) from a total of 22 infected rodents from three hilly villages and then performed molecular analyses [1]. One included publication in our manuscript used the hatching method in the field [2]. As clear water and suitable warm temperature are the pre-requirements to perform the hatching method, unless miracidia are sampled and collected, investigators often use dissection to observe if there are any granulomas with viable eggs in livers of rodents.

Rodent density was measured as the number of rodents captured/the number of the rodent traps. Please see Line 228-229.

We presented a strong sense in emphasizing the potential role of wild rodents in schistosomiasis transmission in order to emphasize our, and others, concern that the role of such small and abundant animals in local transmissions of China (and beyond) have been long ignored. Indeed, in the new revised version (GB 15976-2015) of criteria for schistosomiasis control and elimination in China [3] there is no index regarding infections in any wild mammals, although over 40 mammal species are potential reservoirs for the parasite [4]. Furthermore, we are aware that the potential role of rodents for ongoing transmission is now being considered at WHO levels, and hence is an issue of high topical interest and importance. We have, as above, clarified the viability of schistosomes from rodents from those studies available to date – where miracidia have been successfully hatched (and often also genotyped) from rodents within China. Likewise we also refer to recently published literature showing also matched genotypes of schistosomes circulating between humans and rodents in Africa too (*S. mansoni* in that case [5]). Thus, we have both provided further evidence here to strengthened the support of our argument, that potential ongoing transmission via rodents cannot be ignored, particularly in the elimination era, whilst also acknowledging the inherent limitations of the data available for meta-analyses here to date and the imperative need for future research in this area. We hope you find our compromise acceptable. Please see Lines 6, 66-67, and 466-468.

Reference

1.	Lu DB, Wang TP, Rudge JW, Donnelly CA, Fang GR, Webster JP. Genetic diversity of *Schistosoma japonicum* miracidia from individual rodent hosts. Int J Parasitol. 2011;41(13-14):1371-6. PubMed PMID: 22051401.

2.	Lu C, Zhou LY, Xing XY, Lin DD, Chen T, Chen R, et al. Analysis of transmission risk factors of schistosomiasis in a hilly demonstration plot of transmission interruption. Chin J Parasitol Parasit Dis. 2018;36(04):333-9.

3.	Xu J, Li SZ, Chen JX, Wen LY, Zhou XN. Playing the guiding roles of national criteria and precisely eliminating schistosomiasis in P. R. China. Chin J Schisto Control. 2017;29(1):1-4.

4.	He YX, Salafsky B, Ramaswamy K. Host-parasite relationships of *Schistosoma japonicum* in mammalian hosts. Trends in parasitology. 2001;17(7):320-4. PubMed PMID: 11423374.

5.	Catalano S, Leger E, Fall CB, Borlase A, Diop SD, Berger D, et al. Multihost Transmission of *Schistosoma mansoni* in Senegal, 2015-2018. Emerging infectious diseases. 2020;26(6):1234-42. doi: 10.3201/eid2606.200107. PubMed PMID: 32441625.

As China nears elimination of schistosomiasis and the prevalence in humans and bovines is extremely low approaching zero (see Reference 8 they quote), if rodents play a key role in transmission, why aren’t these prevalence values higher?

Answer: As recently stated within another paper, ‘while praziquantel may have helped reduce prevalence, our evidence suggests that the structural changes and improvements in living conditions had the biggest impact on schistosomiasis in this community’ [6]. Infections in humans and bovines in an endemic area could be much lower if they have less or no contact with infectious waters due to environment change or living condition improvement, which indeed have been occurring across China and all over the world. But in areas where infected wild mammals and intermediate host snails exist, the local transmission could be maintained. Such endemic areas do exist in China [7, 8]. Thus, we are not implying in any way that rodents have been ‘the’ key factor in schistosomiasis transmission across China in general, but instead that ongoing transmission within these rodent wildlife reservoirs, whilst the overall levels have been reduced in humans and livestock, could have the potential to help explain the persistence of schistosomiasis in some areas despite impressive and extensive levels of multisectoral control implemented and also the re-emergence of schistosomiasis within previously ‘cleared/eliminated’ areas.

We fully appreciate raising the potential role of ongoing reservoirs of infection to seed areas of continued transmission and/or re-emergence, particularly those involving notoriously difficult to manage wildlife reservoirs, will not be welcomed to all. However, we also strongly believe that it would be wholly inappropriate to ignore the evidence available to date, even if, as we acknowledge here, currently limited. At very least we hope studies such as ours here will help to raise awareness and will hopefully help stimulate further comprehensive research in this critically important area of research as we progress together through the ‘elimination era’. As above, we have rephrased our text accordingly to emphasize the limitation of some of the data available to date, toned down both our conclusions and the manuscript title itself, but the evidence of a potentially critical trend here, and hence the imperative need for careful acknowledgement and further research in this area, we strongly feel should still be included.

Reference

6.	Silva LK, Barbosa LM, Kovach JD, Dos Santos Teixeira R, Soares ES, Cardoso CW, et al. The changing profile of schistosomiasis in a changing urban landscape. Int J Parasitol. 2020;50(1):27-34. doi: 10.1016/j.ijpara.2019.10.003. PubMed PMID: 31783024; PubMed Central PMCID: PMC6964254.

7.	Liu XP, Wang TP, Wang QZ, Yin XM. Infection status of sources of schistosomiasis japonica in marshland and hilly regions. Journal of Pathogen Biology 2013;8(05):445-7, 10.

8.	Shi HP, Lu DB, Shen L, Shi T, Gu J. Single- or mixed-sex *Schistosoma japonicum* infections of intermediate host snails in hilly areas of Anhui, China. Parasitol Res. 2014;113(2):717-21. PubMed PMID: 24292605.

This reviewer suggests a major rewrite of the article just emphasising the valuable retrospective information presented in Table 1 (indicating the flaws in many of these papers as parameters such as intensity levels, viability/hatchability of eggs were not considered) and de-emphasising the impact on China's path to schistosomiasis elimination.

Answer: Thank you very much. We have taken all the above comments and suggestions into consideration, and have now tuned down our main conclusions and title, whilst still stressing their highly topical potential importance and interest, which we hope is now fully acceptable. Please see, for example, Lines 6, 66-67, and 466-468.
