# Supplementary material for: Meta-analyses of Schistosoma japonicum infections in wild rodents across China over time indicates a potential challenge to the 2030 elimination targets
Source: PLoS Negl Trop Dis. 2020 Sep 2;14(9):e0008652. doi: 10.1371/journal.pntd.0008652 (PMC7491725; doi:10.1371/journal.pntd.0008652)
Supplement: Attachment — Submitted filename: V4 Responses to Reviewers comments_For R3_Clean_Submitted.docx [file pntd.0008652.s006.docx]

Dear Prof. Darren J. Gray and Prof. Justin Remais,

Thank you and your referees very much indeed for the further comments and suggestions on our manuscript (No. **PNTD-D-19-01947R2** entitled ‘Meta-analyses of *Schistosoma japonicum* infections in wild rodents across China over time indicates a potential challenge to the 2030 elimination targets’), all of which we found very helpful and have been incorporated into our further revised text – precise details of which are provided in our *Responses to Referees* below. As suggested, we have now added both discussion and evidence regarding viable infections in rodents, together with further details of our pooled prevalence within the meta-analyses. We have further acknowledged and emphasized the limitation of potential publication bias (few studies included in certain subgroups), which is generally inherent in all meta-analyses. However, our work involves 37 publications with 61 studies included, and presents, for the first time, the pooled infection prevalence in rodents across China. We firmly believe that this could prove useful for all health and policy makers who have the keen interest of the potential role of rodent reservoirs for ongoing/re-emerging schistosomiasis transmission, and also help stimulate the need for more epidemiological and molecular research on schistosome infections in wild animals and their roles in transmission.

We thus hope our re-revised manuscript is now suitable for publication in *PLoS Neglected Tropical Diseases* and we very much look forward to hearing from you.

Kindest regards,

Prof. Da-Bing Lu, on behalf of all the authors

Thanks for toning down the manuscript and for addressing the reviewer comments. I have some additional comments:

1. I think it is import that there is explicit discussion on the ability of rodents to produce viable eggs. you rebutted the reviewer but this needs to go in the manuscript. The discussion should be along lines of do rodents produce schistosome eggs that hatch into infectious miracidia - this should be supported with evidence.

Answer: Thank you very much for your positive comments. As regards the ability of rodents to produce viable eggs, we fully agree and have now explicitly added this into our further revised discussion and provided published evidence to support this (for example, where infection intensity was measured as number of successfully hatched miracidia per gram of faeces [1, 2], and where schistosome genotypes were matched between rodents and other hosts through typing of viable miracidia [3, 4]). Please see Line 400-415. We do also acknowledge that further work is required in this area and again hope the publication of our paper helps stimulate this.

References

1.	Mao SB. Schistosome biology and control of schistosomiasis. Beijing: Publishing House of People's Health; 1990.

2.	Lu DB, Wang TP, Rudge JW, Donnelly CA, Fang GR, Webster JP. Contrasting reservoirs for *Schistosoma japonicum* between marshland and hilly regions in Anhui, China-a two-year longitudinal parasitological survey. Parasitology. 2010;137(1):99-110.

3.	Lu DB, Rudge JW, Wang TP, Donnelly CA, Fang GR, Webster JP. Transmission of *Schistosoma japonicum* in marshland and hilly regions of China: parasite population genetic and sibship structure. PLoS Negl Trop Dis. 2010;4(8):e781. PubMed PMID: 20689829.

4.	Catalano S, Leger E, Fall CB, Borlase A, Diop SD, Berger D, et al. Multihost Transmission of *Schistosoma mansoni* in Senegal, 2015-2018. Emerging infectious diseases. 2020;26(6):1234-42. doi: 10.3201/eid2606.200107. PubMed PMID: 32441625.

2. Greater detail on the pooling of prevalence across the studies needs to be provided in the methods. As it stands in tables 2 & 3 with the provision of total number of rodents and total infected rodents the prevalence does not add up - this is misleading and so the pooled prevalence requires greater explanation

Answer: We apologize for the confusion here. Pooled prevalence (and CIs) with meta-analysis was obtained via aggregation of the results across the multiple studies. This aggregation is not just the simple sum of the data obtained from all the studies, but a procedure that carefully ‘weights’ the results of each study according to its precision. The precision of each study is estimated on the width of the dispersion (i.e. variance), and then the weight of each study is given by the inverse of the variance. In other words, if a study has a wide variance it will have a small weight in determining the final result of the analysis (the pooled prevalence of the meta-analysis), while a study with a small variance will have a greater weight [5]. We here use the method of double arcsine transformation which can address both the problem of confidence limits when calculated possibly outside the 0..1 range and that of variance instability caused by any prevalence of close to 0 or 1 [6]. We have further clarified these details within our revised text (please see Line 219-229) and Table 2 & 3 legends.

We have further checked all data and corrected a few typographical errors, for which we apologize. Please see Table 2. We here kept all prevalences and P values with two decimal places. Thank you very much again for your careful reading.

References

5.	Leandro G. Meta-analysis in Medical Research - The handbook for the understanding and practice of meta-analysis: Blackwell Publishing Ltd; 2005.

6.	Barendregt JJ, Doi SA, Lee YY, Norman RE, Vos T. Meta-analysis of prevalence. Journal of epidemiology and community health. 2013;67(11):974-8. doi: 10.1136/jech-2013-203104. PubMed PMID: 23963506.

3. Greater discussion is required around the increase of prevalence between the 2 time periods in the hill and mountainous region. The prevalence was zero in the 1980 - 2003 period and this is what is driving the significant increase seen in 2004 - 2018. Is this zero estimate correct? significant transmission was ongoing in the 1980-2003 period and I find it strange that the prevalence is zero, noting it is 8% in the lake and marshlands during the same period. only 8 studies were undertaken could it be that this was not enough to capture a true estimate of prevalence. This requires considerable discussion as this result underpins your argument. the same applies to Hubei.

Answer: Thank you very much for your critical and positive comments. As shown below, among eight studies performed in the hilly and mountainous regions in 1980-2003, three reported infections in rodents with infection prevalence ranged between 0.1608% and 0.8696%. The pooled prevalence with meta-analysis is actually estimated to be 0.00026267% (0, 0.0688956%), and to maintain consistency in terms of figures in Table 3, written as 0.00 (0.00, 0.07%). We have however, added additional details to the legend on Table 3 to clarify this.

We are aware that having small numbers of studies could potentially result in a bias in our inferred low estimates. However, having fewer studies performed on this subject could, to a large extent, reflect the local professional awareness (or lack of) in schistosomiasis control. We have now added further discussion on this. Please see Line 369-380. We have also further mentioned the limitation of potential publication bias, which, as mentioned, is inherent in all meta-analyses. Please see Line 436-438.
